# Evaluation of Antioxidant and Enzyme Inhibition Properties of *Croton hirtus* L’Hér. Extracts Obtained with Different Solvents

**DOI:** 10.3390/molecules26071902

**Published:** 2021-03-28

**Authors:** Stefano Dall’Acqua, Kouadio Ibrahime Sinan, Stefania Sut, Irene Ferrarese, Ouattara Katinan Etienne, Mohamad Fawzi Mahomoodally, Devina Lobine, Gokhan Zengin

**Affiliations:** 1 Department of Pharmaceutical and Pharmacological Sciences, University of Padova, Via Marzolo 5, 35131 Padova, Italy; stefania_sut@hotmail.it (S.S.); irene.ferrarese@unipd.it (I.F.); 2 Department of Biology, Science Faculty, Selcuk University, Campus, 42130 Konya, Turkey; sinankouadio@gmail.com (K.I.S.); gokhanzengin@selcuk.edu.tr (G.Z.); 3 Laboratoire de Botanique, UFR Biosciences, Université Félix Houphouët-Boigny, 00225 Abidjan, Côte d’Ivoire; katinan.etienne@gmail.com; 4 Department of Health Sciences, Faculty of Medicine and Health Sciences, University of Mauritius, 230 Réduit, Mauritius; devinalobine@gmail.com

**Keywords:** *Croton hirtus*, enzyme, flavonoid glycosides, bioactive agents

## Abstract

*Croton hirtus* L’Hér methanol extract was studied by NMR and two different LC-DAD-MS^n^ using electrospray (ESI) and atmospheric pressure chemical ionization (APCI) sources to obtain a quali-quantitative fingerprint. Forty different phytochemicals were identified, and twenty of them were quantified, whereas the main constituents were dihydro α ionol-*O*-[arabinosil(1-6) glucoside] (133 mg/g), dihydro β ionol-*O*-[arabinosil(1-6) glucoside] (80 mg/g), β-sitosterol (49 mg/g), and isorhamnetin-3-*O*-rutinoside (26 mg/g). *C. hirtus* was extracted with different solvents—namely, water, methanol, dichloromethane, and ethyl acetate—and the extracts were assayed using different in vitro tests. The methanolic extracts presented the highest 1,1-diphenyl-2-picrylhydrazyl (DPPH), 2,2′-azino-bis(3-ethylbenzothiazoline)-6-sulfonic acid (ABTS), and ferric reducing antioxidant power (FRAP) values. All the tested extracts exhibited inhibitory effects on acetylcholinesterase (AChE) and butyrylcholinesterase (BChE), with a higher activity observed for dichloromethane (AChE: 5.03 and BChE: 16.41 mgGALAE/g), while the methanolic extract showed highest impact against tyrosinase (49.83 mgKAE/g). Taken together, these findings suggest *C. hirtus* as a novel source of bioactive phytochemicals with potential for commercial development.

## 1. Introduction

The genus, *Croton* (Euphorbiaceae) comprises of about 1300 species, mostly widespread in tropical and subtropical regions of both hemispheres [1]. Several members of the genus are popular in folk medicine as they are commonly used against a range of illnesses such as inflammatory conditions, pain, diabetes, hypertension, malaria, gastrointestinal disturbances, and ulcers, amongst others [1,2,3]. *Croton cajucara* Benth., *C. celtidifolius* Baill., *C. eluteria* Bennett, *C. zambesicus* Müll. Arg., and *C. macrostachys* Hochst. ex Rich are some of the *Croton* species used for medicinal purposes. The chemistry of the genus is considerably diverse. Diterpenoids such as phorbol esters, clerodane, neoclerodane, isopimarane, kaurane, and labdane type terpenoids are the principal bioactive components in the genus. Some *Croton* species are also rich in alkaloids such as taspine and julocrotol [1,4].

*Croton hirtus* L’Hér., also known as *C. glandulosus*, is an erect shrub, and various studies have investigated the chemical composition of its essential oil, leaves, and roots. In particular, Daouda et al. [2] have reported the antimicrobial action of *C. hirtus* essential oil, which was found to contain 93.49% of terpene derivatives, with 15.55% monoterpenes and 77.94% sesquiterpenes. In a study by de Lima, Medeiros, Cunha, da Silva, de Andrade, Neto, Lopes, Steffen, Araújo, and Reis [3], the presence of spathulenol (26.7%), *E*-caryophyllene (10.0%), bicyclogermacrene (9.5%), α-cadinol (7.7%), and cubenol (7.0%) as main constituents of the essential oils extracted from *C. hirtus* leaves was reported. In 2019, Rosandy, Azman, Khalid, Othaman, Lazim, Choudary, Syah, Latip, Said, and Bakar [4] identified two new diterpenoids from the roots of *C. hirtus.* Preliminary reports suggested the presence of alkaloid, anthraquinone, flavonoid, steroids, and terpenoid in different parts of *C. hirtus* [5,6]. However, as literature shows, information available related to the phytoconstituents of the plant are punctual, and a complete accurate study of C. *hirtus* leaves composition is still missing. Moreover, there is a scarcity of information in literature on pharmacological properties of *C. hirtus*. In a recent paper, Kim et al. [7] reported the anti-inflammatory effects of *C. hirtus* via inhibition of nuclear factor kappa-light-chain-enhancer of activated B cells (NF-κB) signaling pathway.

In this study, detailed phytochemical fingerprinting of *C. hirtus* was obtained using NMR analysis of the methanol extract and LC-DAD-MS^n^ methods, one using electrospray ionization (ESI) and one using atmospheric pressure chemical ionization (APCI) ion sources.

Once establishing the chemical composition to assess biological activities, plant material was extracted with different solvents—namely, water, methanol, dichloromethane, and ethyl acetate—and the obtained extracts were subjected to different in vitro bioassays. Free radical scavenging, reducing power, metal chelating, and phosphomolybdenum assays were employed for evaluating antioxidant effects of the different extracts, while the enzyme inhibitory activity against targeted enzymes such as cholinesterases, tyrosinase, α-amylase, and α-glucosidase—involved in significant human diseases—were determined. As a general measurement of the content of extracted constituents, the phenolics content of *C. hirtus* were determined by spectrophotometrical assays.

## 2. Results and Discussion

### 2.1. Phytochemical Analysis

**Figure 1:**
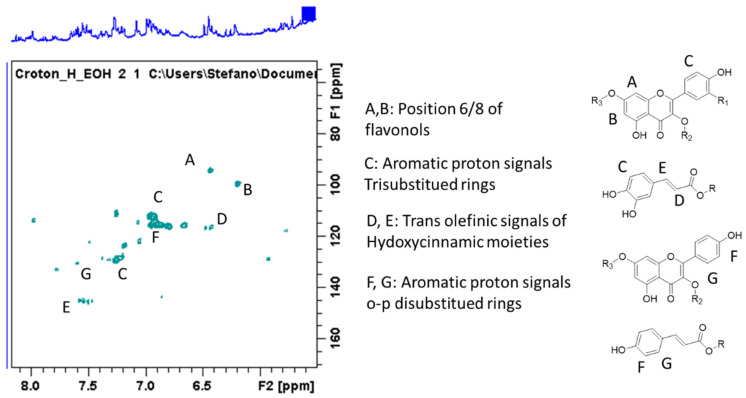
Heteronuclear single quantum coherence- Distortionless enhancement by polarization transfer, (HSCQ-DEPT) portion of the spectrum showing the signals ascribable to phenolic derivatives.

**Figure 2:**
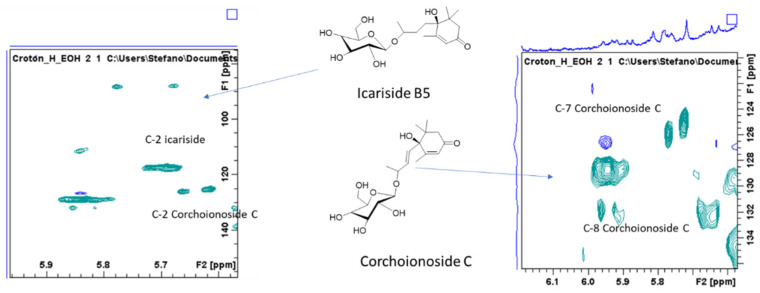
HSQC-DEPT portion of spectrum showing some of the diagnostic signals used to confirm structure identification.

C. *hirtus* methanol extract was subjected to different NMR experiments as heteronuclear single quantum coherence (HSQC) and heteronuclear multiple bond correlation (HMBC) as well as correlation spectroscopy (COSY). Based on the chemical shifts of ^1^H and ^13^C and the correlations in the 2D spectra, some tentative assignments were obtained and are summarized in Table 1. The presence of simple phenolics such as protocatechuic acid were tentatively established due to the presence of spin system relating to aromatic signals observed in COSY, suggesting the presence of 1,3,4 tri-substituted ring, as well as on the basis of the chemical shift of carbons (obtained from the HSQC) and the long-range correlation with carboxyl function (Table 1; Figure 1). Further signals can be ascribed to hydroxycinnamic acid derivatives due to the olefinic signals at δ 7.59 and 6.45, as well as due to HMBC correlations with aromatic positions. Signals suggesting the presence of flavonoid derivatives can be also observed—namely, the H-6 and H-8 position of flavonols as well as signals ascribable to ring A and B (Table 1; Figure 1). Additionally, different signals support the presence of sugar and glycosidic derivatives. In the 1D and 2D NMR spectrum, some signals are ascribable to megastigmane derivative (Table 1), which is consistent with the literature [8]. In particular, the diagnostic signals are the geminal methyl groups that are partially overlapped with other aliphatic signals in the extract and the sp^2^ olefinic protons of the HSQC-DEPT spectrum. Compounds structures were proposed as icariside B and corchionoside C (Figure 2), and an enlargement of the HSQC is reported with some key signals highlighted. Unsaturated proton signals of the α ionol derivative include the one at δ 5.25 (δ_C_ 126.2) that shows HMBC correlations with carbon signals at δ_C_ 51.0 (CH in the six membered rings linked to the side chain), at δ_C_ 36.0 (CH_2_), and at δ_C_ 135.1 (the quaternary carbon signal). From the methyl group at δ 2.05, correlations are observed with the carbon at δ 135.1 and with 126.5. Secondary methyl group at δ 1.14 can be assigned to the methyl group nearby the glycosidic linkage due to the HMBC with carbon at δ 73.1. These data and the signals largely observed in the sugar region supported the MS identification of compound dehydro α ionol-*O*-[arabinosil(1-6) glucoside]. Diagnostic signals for the isomer dehydro β ionol-*O*-[arabinosil(1-6) glucoside] are the CH sp2 and by the HMBC with the CH at δ 72 and the secondary methyl group.

**Figure 3:**
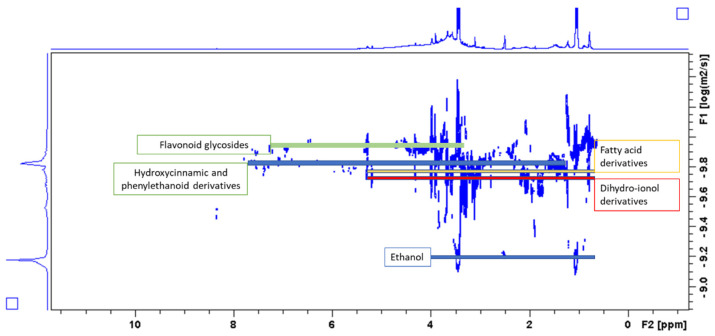
Diffusion order spectroscopy (DOSY) spectrum showing some of the different classes of constituents present in the *C. hirtus* extract.

A diffusion order spectroscopy experiment (DOSY) was also performed to observe different behavior of the various extracted phytochemicals present in the mixture. As shown in Figure 3, the spectrum has a group of signals that can be distinguished on the basis of their diffusion coefficients. Ethanol presents its signals largely separated from the other belonging to the plant constituents. The red line indicates the diffusion coefficient of dihydro ionol derivatives, the yellow one shows group of signals that can be ascribed to fatty acid derivatives, while hydroxycinnamic/phenylethanol compounds as well as flavonoid glycosides present larger values of diffusion coefficients.

**Figure 4:**
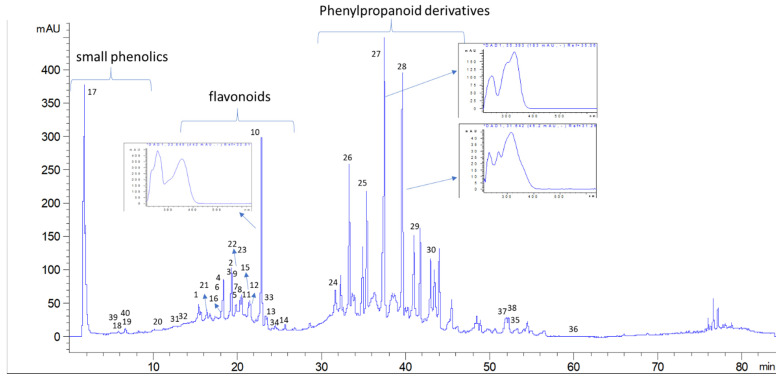
LC-DAD chromatogram (330 nm) showing the peak group assigned to small phenolics, flavonoids, and phenylpropanoid derivatives; exemplificative UV spectra of the peaks are included. Peaks numbers refer to Table 2.

To have detailed quali-quantitative fingerprint, liquid chromatography coupled with diode array detector and mass spectrometry (LC-DAD-MS^n^) methods were developed, and to detect a larger number of the different constituents present in the plant, electrospray (ESI) and atmospheric pressure chemical ionization (APCI) ion sources were used. Phytoconstituents were studied based on MS fragmentation and on the UV spectra (for the phenolic compounds). An exemplificative chromatogram is reported in Figure 4.

The LC-DAD chromatogram obtained for the methanol extract showed a series of peaks presenting typical UV spectrums ascribable to flavonoids, hydroxycinnamic acids and small phenolics as represented in Figure 1. Mass spectrometric data were acquired both in negative and in positive ion mode using electrospray source, and the study of MS^n^ fragmentation allowed the identification of several derivatives that were quantified based on the diode array chromatographic traces using reference compounds.

**Figure 5:**
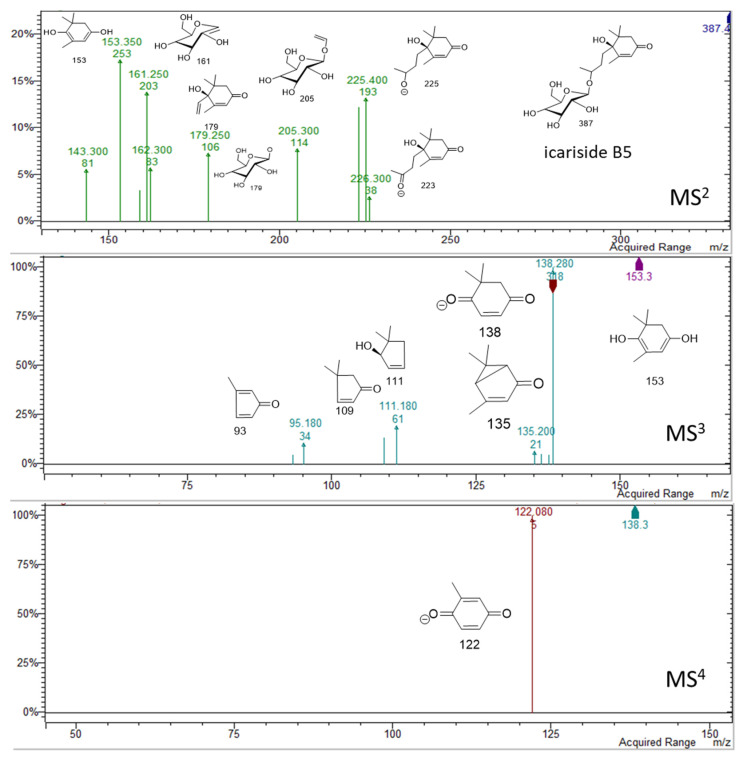
Spectral and fragmentations of icariside B5: MS^2^ from parent ion 387 *m/z*, MS^3^ from fragment 153 *m/z*, MS^4^ from fragment 138 *m/z.*

**Figure 6:**
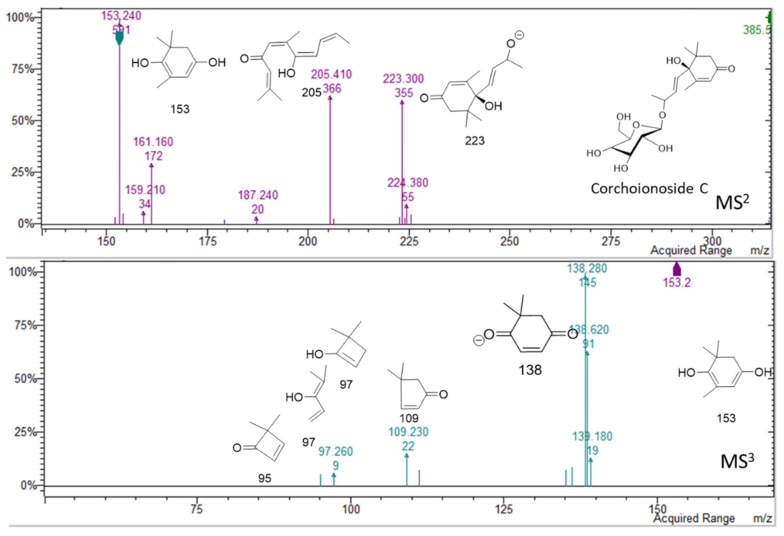
Spectra and fragmentations proposed for corchoionoside C: MS^1^ from parent ion 385 *m/z*, MS^2^ from fragment 153 *m/z.*

Phenolic compounds were abundant in the plant methanol extract. Some small phenolics were detected in the first minutes of the chromatograms. Peaks were assigned to caffeic acid hexoside **(17)** [9], protocatechuic acid hexoside **(18) [9]**, and benzyl alcohol hexose pentose **(19)** [10]. The contents of these simple phenolics in the extract was 19 mg/g of dry extract (Table 2). In the region of 10–13 min in the chromatogram, no significant peaks were detected at wavelength at DAD detector. Nevertheless, two peaks at retention times of 13.1 and 13.8 min were observed in negative mode, suggesting the presence of compounds that lack chromophore (Figure in Supplementary Materials). These peaks present adducts with formic acid [M + HCOOH − H]^−^ at *m/z* 433 and 431 and also deprotonated molecular ions [M − H]^−^ at *m/z* 387 and 385, respectively. The fragmentation showed the loss of a hexose unit, followed by the loss of the megastigmane moiety [11,12]. On the basis of the fragmentation schemes, the peak at min 13.1 was assigned to icariside B5 **(31)** (*m/z* 387) [13], while the peak at 13.9 min was assigned to corchoionoside C **(32)** (*m/z* 385). Spectra and proposed structures of main fragments for the two derivatives are summarized in Figure 5 and Figure 6.

**Figure 7:**
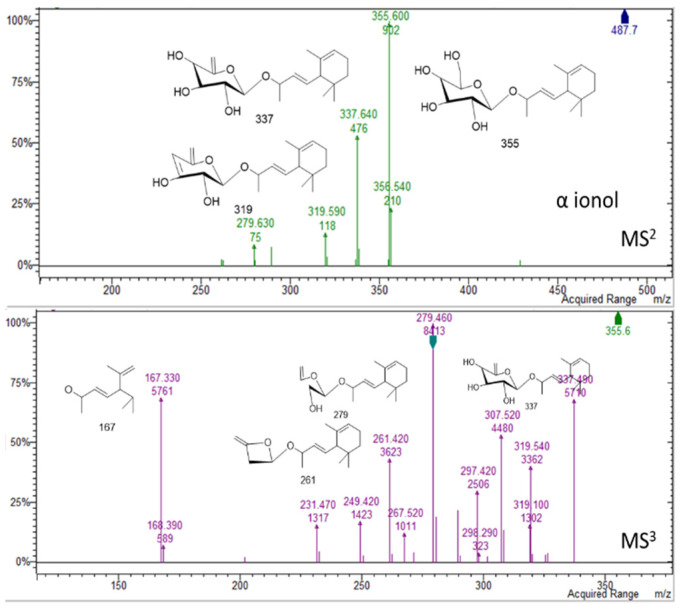
Mass spectral details of dihydro α ionol-*O*-[arabinosil(1-6) glucoside].

**Figure 8:**
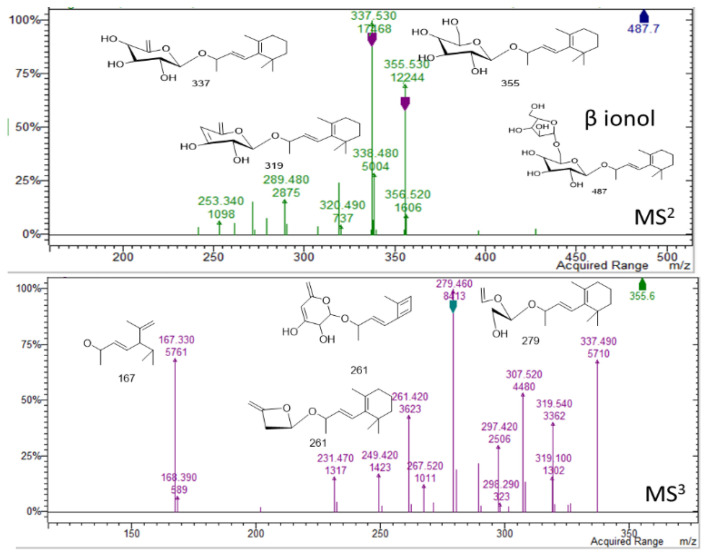
Mass spectral details of dihydro β ionol-*O*-[arabinosil(1-6) glucoside].

In the region of 16–25 min peaks showing UV spectrum typical of flavonols, characterized by maximum absorption in the range of 350 and 265 nm (Figure 1) [9], were detected. The NMR data showed signals ascribable to flavonoid derivatives as summarized in Table 1, and the main signals related to aromatic portion are indicated in Figure 4. Kaempferol and quercetin as aglycones can be observed due to the MS^n^ fragmentation spectra [14]. Glycosylation position can be tentatively assigned based on the relative intensity of aglycone fragments [14,15,16]. The amount of flavonoids and their glycosides **(1–16)** was 96 mg/g of dried extract. Isorhamnetin-3-*O*-rutinoside **(10),** kaempferol-3-*O*-hexosil-deoxyhexoside **(15)**, and quercetin-3-apiofuranosyl-glucopiranoside **(6)** were the most abundant derivatives with 25.91, 18.0, and 16.13 mg/g extract, respectively. LC-MS^n^ data in negative ion mode, in the region of 23–26 min of the chromatogram, revealed two intense peaks (see Supplementary Material Figure S3) that present [M − H]^−^ at *m/z* 487. The fragmentation pattern of these two derivatives is reported in Figure 7 and Figure 8 and can be ascribed to two glycosidic derivatives of ionones, based on the comparison with databases, and thus the compounds were identified as dihydro α ionol-*O*-[arabinosil(1-6) glucoside] and the dihydro β ionol-*O*-[arabinosil(1-6) glucoside]. Their amounts are notable in the extract being 132 and 79 mg/g extract, respectively. Furthermore, different peaks observed in the chromatogram region from 32 to 50 min present UV spectra ascribable to hydroxycinnamic acids. The NMR data confirmed the presence of different units of hydroxycinnamic derivatives due to multiple doublet signals ascribed to the double bond of these compounds. Several peaks can be ascribed to phenylethanoid glycosides (Table 1, Figure 4). At retention time 19.4 min, the MS^n^ fragmentation scheme suggests that the structure can be tentatively assigned to leonoside A (*m/z* 769) **(22),** while at 20.4 min peak presenting [M − H]^−^ at *m/z* 755 can be annotated as forsythoside B **(23)** [17]. Two derivatives presenting [M − H]^−^ at *m/z* 797 are annotated as ferrusinogide C **(25)** and its isomer (**24).** Several peaks present adduct with formic acid [M + HCOOH − H]^−^ at *m/z* 856 and [M − H]^−^ at *m/z* 810. This compound presents fragmentation MS^3^ with a very strong peak at *m/z* 601 and which further forms fragment ions at *m/z* 487, 469, 451, and 337. The latter fragment suggests that they are feruloyl quinic acid derivatives. The UV spectrum confirms the presence of a hydroxycinnamic unit, and the fragmentation scheme indicates the presence of triterpene unit or a big portion of sugar, but due to absence of a similar pattern of fragmentation in the literature, we preferred not to assign any structure to those peaks. As these compounds were not identified, they were not included in the table; however, their isolation is in progress.

Further compounds presenting molecular ion [M − H]^−^ at *m/z* 663 were assigned to ester of sucrose with ferulic acid and *p*-coumaric acid. Five different isomers **(26–30)** were observed, probably due to the ester linkages at different positions. From MS spectra, it is not possible to assign the different isomers due to lack of specific literature.

In the extract, the overall amount of phenyl propanoid and phenylethanoid derivatives is 100 mg/g. At 53.4 min, kongensin D **(35)**, a diterpenoid derivative, was identified based on *m/z* value at 331. Phytosterols were detected using APCI ion source operating in positive ion mode, and β-sitosterol **(36)** was the most abundant compound. The results of the fingerprinting showed that the extract contains different classes of compounds, such as phytosterols, flavonoid glycosides, hydroxycinnamic derivatives, megastigmane, and other terpenoids, indicating that *C. hirtus* can be considered as a valuable source of bioactive constituents.

Due to the complex nature of constituents detected in the plant, we decided to perform extraction using water and organic solvents (dichloromethane (DCM)), ethyl acetate (EA), and methanol (MeOH)). Different in vitro assays were then performed using the obtained extracts. The results are illustrated in Table 3. A notable amount of total phenolic content was recorded for all the extracts, ranging from 17.96 (MeOH) and 24.24 (DCM) mg GAE/g extract. A significant difference in total flavonoid content using different solvents was observed. Among the solvents used, the MeOH extract (50.16 mg RE/g), followed by EA extract (29.28 mg RE/g extract), resulted in the highest total flavonoid content using the spectrophotometric assay. The variation in the yields can be related to the different solvent polarities and to the different solubility and polarity of the phytoconstituents [18,19]. Such parameters can also be influenced by the ability of the different solvents to penetrate the plant tissue and to solubilize the classes of compounds.

### 2.2. Antioxidant Ability

Oxidative stress is a phenomenon that arises when reactive oxygen species overwhelms the intrinsic antioxidant defense mechanism. Considerable evidence implicates oxidative stress in the pathophysiology of many diseases and associated complications including diabetes, cardiovascular diseases, and dementia [20,21]. As synthetic antioxidants such as butylated hydroxytoluene (BHT) and butylated hydroxyanisole (BHA) have been reported to exert negative effects on human health, there is a renewed interest to find novel antioxidant compounds with more efficacy and minimal or no side effects. In this context, medicinal plants in particular have been widely studied for discovering potential novel antioxidant molecules [22]. Some of *Croton* species that have been reported for their antioxidant capacity include *C. celtidifolius* Baill. [23], *C. lechleri* Muell.-Arg. [24], *C. zambesicus* Muell.-Arg. [25], and *C. cajucara* Benth. [26].

Therefore, this investigation also endeavored to evaluate the antioxidant potential of the different extracts of *C. hirtus* by employing different in vitro bioassays—namely, free radical scavenging (DPPH and ABTS), reducing power (CUPRAC and FRAP), phosphomolybdenum, and ferrous-ion chelating assays. As illustrated in Table 4, different extracts possessed varied free radical scavenging and reducing capacities. The results of DPPH and ABTS assays indicated that the water extract displayed the highest free radical scavenging ability, with a mean value of 41.08 and 64.84 mg TE/g extract, respectively. For CUPRAC assay, the DCM (88.67 mg TE/g extract) extract, followed by water (78.17 mg TE/g extract) extract, showed highest reducing power. The order of the FRAP assay was water > MeOH > DCM > EA. This indicates that the hydrophilic compounds in the extract are, in general, mostly involved in the antioxidant effect in the assays’ conditions. Considering the fingerprinting chemical analysis, these effects can be probably ascribed to hydroxycinnamic and flavonoid derivatives. This observation is in agreement with a previous report that demonstrated that highly polar solvents, such as water and methanol, have a high effectiveness as antioxidants [22]. In the literature, we observed different results for different solvent extracts [27,28]. For example, although polar extracts had the highest levels of phenolic compounds in some studies, apolar extracts exhibited stronger free radical and reducing abilities. This fact can be explained by different reasons. For example, the spectrophotometric assays could have some drawbacks, and not only phenolics but also other classes of compounds could play a role. Furthermore, reaction conditions, organic solvent, aqueous buffer, and pH influence the experimental response of the mixture and compounds, leading to different effects in the antioxidant reactions. Altogether, the utilization of more assays could provide accurate information of the antioxidant capacity of plant extracts.

The phosphomolybdenum assay was employed to estimate the total antioxidant capacity of the *C. hirtus* extracts. The DCM extract was found to show the highest total antioxidant capacity, with a mean value of 2.70 mmol TE/g extract. In the case of ferrous-ion chelating activity, the EA (18.26 mg EDTAE/g extract) and water (17.94 mg EDTAE/g extract) extracts showed the highest activity. In total, despite of its lower total phenolic and flavonoid contents, the water extract showed considerably higher antioxidant capacity as compared with other tested extracts. Such results suggest lipophilic fraction has a role in mediating the antioxidant effects, and some effect can also be ascribed to fatty acid and phytosterols that have been reported as significant antioxidant compounds [29,30,31].

In the literature, the extracts of some *Croton* species and their isolated compounds exhibited remarkable antioxidant properties. For example, Aderogba et al. [25] isolated three compounds from *C. zambesicus* and one of them displayed good DPPH radical scavenging ability with an IC_50_ value of 200 µM. Similarly, Azevedo et al. [26] reported significant radical scavenging abilities of *C. cajucara* isolated compounds. When combining all data on the genus *Croton*, they could be considered as a significant source of natural antioxidants.

### 2.3. Enzyme Inhibitory Ability

Due to their essential catalytic role in several physiological processes, enzymes are considered as prime targets for drug design for alleviating diseases. Indeed, the therapy of some important human aliments—namely, hypertension, diabetes, inflammatory and neurodegenerative diseases—includes the use of enzyme inhibitors. For instance, α-amylase and α-glucosidase enzymes are the targeted enzymes in diabetes, while cholinesterases (acetylcholinesterase and butyrylcholinesterase) are the known therapeutic targets in Alzheimer’s disease [32,33].

The inhibitory action of the studied *C. hirtus* extracts against cholinesterase, tyrosinase, α-amylase, and α-glucosidase were determined. Table 5 illustrates the inhibitory potential of the different extracts against the studied enzymes, expressed as equivalent values of standards. The tested solvents displayed significantly distinct inhibitory potentials against AChE, with the DCM and EA extracts (5.03 mg GALAE/g extract, 4.84 mg GALAE/g extract) being most potent but with significant effect for methanol (4.05 mg GALAE/g extract). All the extracts showed remarkable anti-BChE effects, with mean values ranging from 16.41 to 15.86 mg GALAE/g extract. This result suggests that different constituents of the plant may exert inhibitory activity on these enzymes. With respect to the composition of the methanol extract, quercetin, kaempferol, and isorhamnetin derivatives detected in the extract have been previously documented as AChE inhibitors [34,35,36]. In particular, quercetin-3-apiofuranosyl-glucopyranoside, isorhamnetin-3-*O*-rutinoside, and kaempferol-3-*O*-hexosil-deoxyhexoside, which are detected in high level in the methanol extracts, could be potentially the main flavonoid derivatives, attributing to the anti-AChE effects. Rutin was also reported by Szwajgier et al. [37] as a significant cholinesterase agent. Furthermore, as previously reported, the hydroxycinnamic acids, such as caffeic acid hexoside and protocatechuic acid hexoside, detected in the extract could be potent AChE inhibitors [34]. For the lipophilic extracts, it can be postulated that the effects were mediated by the presence of phytosterols, particularly β-sitosterol, which have been reported as AChE and BChE inhibitors [38].

All the extracts except the water extract showed considerable tyrosinase inhibition, with values of 49.83, 34.81, and 24.39 mg KAE/g extract for MeOH, EA, and DCM, respectively. Most of the activity can be correlated to hydrophilic phenolic compounds, but as we described for AChE and BChE, a significant role is clearly indicated also by results for the lipophilic constituents. Modest activity against α-amylase activity was exerted by all the extracts (0.14–0.75 nmol ACAE/g extract), whilst only the DCM extract (1.68 nmol ACAE/g) was effective at inhibiting α-glucosidase. Similar to cholinesterases’ inhibitory ability, the other enzyme inhibitory abilities could be attributed to the presence of chemical components identified, including rutin, caffeic acid, and protocatechuic acid [39,40,41,42]. Literature search on the enzyme inhibitory properties of the genus *Croton* resulted in a few studies on the subject. Aderogba et al. [25] reported on the inhibitory properties of flavonols and indol alkaloids from *C. menyharthii* against acetylcholinesterase, and glucosidase and especially alkaloids exhibited significant acetylcholinesterase abilities. Similarly, Shahwar et al. [43] isolated some alkaloids from *C. sparsiflorus*, and most of them had excellent acetylcholinesterase inhibitory properties. Keerthana et al. [44] reported significant amylase inhibition capacity for *C. bonplandianum* ethanolic leaf extract. The remarkable glucosidase inhibitory properties of *C. thurifer* components were also described by Morocho et al. [45]. With all observations, the genus *Croton* could be considered as a source of great potential for natural enzyme inhibitors to combat global health problems.

### 2.4. K-Medoids Clustering

**Figure 9:**
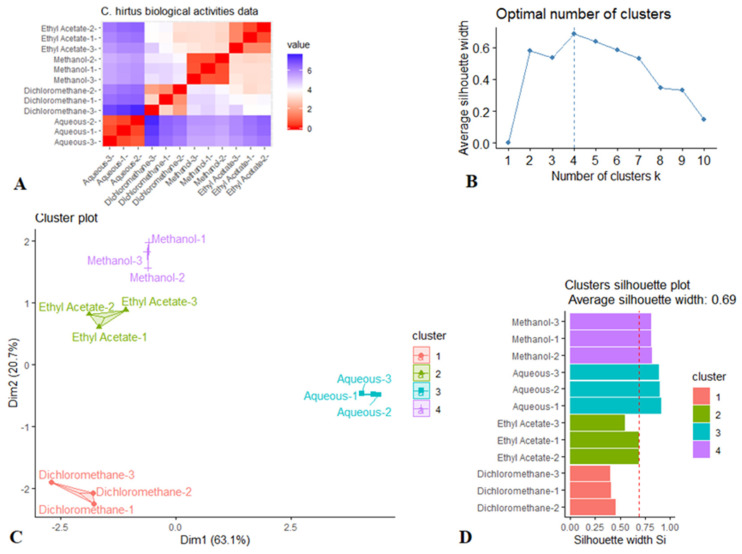
A *k*-medoids clustering analysis of biological activities of *C. hirtus.* (**A**) Distance matrix showing the dissimilarity between each pair of subjects (Red color: high similarity, Blue color: low similarity). (**B**) Number of clusters. (**C**) Scatter plot. (**D**) Silhouette plot displaying the average distance between clusters.

Following the initial screening of biological activities of the prepared extracts by univariate analysis, *k*-medoids cluster analysis was performed with aim of assessing the effects of the solvents used for extraction. The *k*-medoids model is a robust clustering approach for identifying and partitioning a dataset into an optimal number of homogeneous clusters. This approach allowed us to visually evaluate the clustering tendency of the samples from the very beginning. As observed in Figure 9, there is a low similarity among the subjects representing each solvent (Figure 9A). This points out a high intra-class similarity and low inter-class similarity and confirms that there is a cluster structure in the *C. hirtus* biological activities dataset. Based on Figure 9B, it can be observed the optimal number of clusters is 4. Figure 9C depicts the *k*-medoids scatter plot, and it can be observed that the samples were grouped depending on the solvents used and distributed differently in the hyperspace along the two dimensions. Therefore, this statistical approach demonstrated that the biological activities of *C. hirtus* were influenced by the extraction solvents. Notably, the cluster validation was found to be excellent, being 0.69 for the silhouette coefficient, thus confirming the goodness of the *k*-medoids model (Figure 9D).

Many researchers have studied and evaluated the effect of different types of solvents on biological activities of various *Croton* species [1,23,24,25]. These studies indicated that the selectivity of the solvent is crucial not only for obtaining optimum yield of one or more desired molecules but also for pharmacological effects of the resulting molecules. In fact, secondary metabolites possess different degrees of polarity; thereby, the extractive solvent should be chosen carefully to ensure a good dissolution of secondary metabolites intended to be studied. For example, aqueous solvent with a dielectric constant of 80 dissolves polar molecules more rapidly than non-polar solvent hexane due to its low dielectric constant of 1.89.

## 3. Materials and Methods

### 3.1. Preparation of Extracts

Plant samples were collected from Lolobo (Yamoussoukro) of Ivory Coast in 2019 (during summer season) and identified by Ouattara Katinan Etienne (Botanist at Université Félix Houphouet Boigny, Ivory Coast). The plant samples were deposited in the Department of Biology at Selcuk University. The roots were removed and the aerial parts (whole plant with flowers, stems, and foliar tissue) were used. The plant materials were dried in shade for about 10 days and then grounded using a laboratory mill.

In this study, different extraction procedures were employed. As starting point, methanol was used as solvent. At a later stage, solvents with different polarities (dichloromethane, ethyl acetate, water) were also used. Maceration technique was used for dichloromethane, ethyl acetate, and methanol. For this purpose, 5 g of the samples were macerated with respective solvents (individually) for 24 h at room temperature (24 °C ± 1) on a magnetic stirrer, and solvents were changed twice. The mixtures then were filtered and concentrated using a rotary-evaporator. With respect to the water extract, 5 g of the plant material was infused in 100 mL boiled water. Then, the water was dried by using freeze-drying. All the extracts were stored + 4 °C until further studies.

### 3.2. Spectrophotometric Assays for Total Phenolic and Flavonoids

Total phenolic and flavonoid contents of *C. hirtus* extracts were assayed by colorimetric methods according to Zengin and Aktumsek [46]. Gallic acid (GAE) and rutin (RE) were chosen as standards for phenols and flavonoids, respectively.

### 3.3. Fingerprinting of Phytoconstituents by LC-DAD-ESI-MS^n^, LC-DAD-APCI-MS^n^

Dried methanol extract (100 mg) was dissolved in 15 mL of methanol and then subjected to sonication for 15 min at room temperature. The sample was centrifuged at 13,000 rpm, and the supernatant was removed and used for analysis. For the mass spectrometry detection, we used electrospray (ESI) source for phenolics, atmospheric pressure chemical ionization (APCI) ion source for lipophilic compounds as phytosterols and triterpene. Apparatus used was Agilent 1260 chromatograph (Santa Clara, CA, USA) equipped with 1260 diode array detector (DAD) and Varian MS-500 ion trap mass spectrometer equipped with ESI or APCI ion source (Varian inc, Palo Alto, CA, USA). A “T” splitter was installed immediately after chromatographic column allowing the separation of the eluate, half to DAD and half to Agilent/Varian MS-500 ion trap mass spectrometer. UV-Vis spectra were acquired in the range of 190–400 nm.

For the phenolic analysis, synergy RP polar (Phenomenex, Bologna, Italy) was used as stationary phase (3.0 × 150 m^2^; 4 micron) and as eluents, water 1% formic acid (A) and methanol (B) were used. Elution gradient started with 95% of A and went to 10% of A in 50 min, then the amount of B was increased to 100% in 55 min. The flow rate was 400 µL/min. The MS spectra were acquired in negative ion mode in 50–2000 *m/z* range using an ESI source; drying gas temperature started at 295 °C and in 40 min decreased to 250; drying gas pressure was 25 psi; nebulizer was 30 psi; needle was set at 4500 V, RF 85%, capillary 80 V. Quantification of compounds was obtained using DAD. Chlorogenic acid solutions (of concentration ranging from 100 to 1 µg/mL) were used to create calibration curve for the quantification of the different hydroxycinnamic acid derivatives at 330 nm. Calibration curve was Y = 43.325x + 0.236 (*R*^2^ = 0.9989). For the quantification of flavonols, quercetin and kaempferol solutions (range 100–1 µg/mL) were used at 350 nm, and calibration curves were Y = 25.3x + 0.55 (*R*^2^ = 0.9998) and Y = 20.3x + 0.91 (*R*^2^ = 0.9991), respectively. Rutin was used for quantification of quercetin glycosides; isorhamnetin-7-*O*-glucoside and kaempferol-3-*O*-glucoside were used for quantification of isorhamnetin and kaempferol derivatives. Solutions were prepared in the range of 120–0.61 µg/mL, and chromatograms were recorded at 350 nm; calibration curves were Y = 31.62x + 1.021 (*R*^2^ = 0.9993), Y = 33.11x + 0.841 (*R*^2^ = 0.9991), and Y = 23.42x + 1.042 (*R*^2^ = 0.9989) for rutin, isorhamnetin-7-*O*-glucoside, and kaempferol-3-*O*-glucoside, respectively. Benzoic acid was used for protocatechuic acid and small phenolics using solutions (of concentration ranging from 130–1.30 µg/mL) at 280 nm and calibration curve was Y = 81.1x + 0.98 (*R*^2^ = 0.9996). For terpenoids, a mixture of dihydro α ionol-*O*-[arabinosil(1-6) glucoside] and dihydro β ionol-*O*-[arabinosil(1-6) glucoside was purified using an Agilent 1260 chromatograph equipped with analytical fraction collector. As stationary phase, an Agilent XDB C18 3.0 × 150 m^2^ was used, and isocratic elution was used (70% water 0.1% formic acid, 30% methanol) at flow rate of 4 mL/min. Fraction containing compounds were pooled and dried under nitrogen flow and redissolved in deuterated methanol. The amount of α ionol-*O*-[arabinosil(1-6) glucoside] and dihydro β ionol-*O*-[arabinosil(1-6) glucoside] in the mixture was determined using quantitative NMR with a previously published procedure that used caffeine as internal standard [47]. Solvent was then removed and a solution in methanol was prepared and diluted preparing solutions in the range of 300–1.0 µg/mL. Calibration curve was Y = 115.1x + 10.321 (*R*^2^ = 0.9989) using MS detector in negative ion mode.

### 3.4. HPLC-(APCI)-MS Analysis of Phytosterols

For the phytosterol constituents, an Agilent Zorbax Eclipse XDB-C18 column (Agilent, Santa Clara, CA, USA) (3.0 mm × 150 mm, 3.5 µm) was used as stationary phase. All analytical details are described in our previous publications [48]. The compounds were identified based on the comparison with the literature and reference compounds, when available. As standards, known solutions of β-sitosterol (of concentration ranging from 176 to 1.76 µg/mL) and stigmasterol (of concentration ranging from 185.6 to 1.9 µg/mL) were used.

Compound’s quantification was obtained via calibration curve derived from β-sitosterol and stigmasterol solutions. Calibration curves were Y = 0.63x + 0.2705 (*R*^2^ = 0.99611) for β-sitosterol and Y = 2.1153x − 15.216 (*R*^2^ = 0.9949) for stigmasterol.

### 3.5. NMR Analysis

1D and 2D NMR spectra were obtained on a Bruker Avance III 400 Ultrashield spectrometer with 400 MHz magnet. NMR spectra were acquired in MeOD-d4 (Sigma-Aldrich) with TMS as an internal standard. Duran^®^ 4.95 mm NMR tubes (Duran Group, Mainz, Germany) were used. Chemical shifts are expressed in δ values in ppm. ^1^H-NMR and HSQC-DEPT, HMBC, and COSY experiments were acquired using standard Bruker sequences measuring p1 and d1 for each acquired sample. Samples of dried extract were initially dissolved in 100 mg in MeOD-*d_4_* then centrifuged, and the liquid was used for NMR measurements. DOSY experiments were performed using the ledbpgp2s sequence.

### 3.6. Determination of Antioxidant and Enzyme Inhibitory Effects

To detect antioxidant properties, we used several chemical assays including different mechanisms—namely, radical scavenging, reducing power and metal chelating. Trolox (TE) and ethylenediaminetetraacetic acid (EDTA) were used as standard antioxidant compounds. The results are expressed as equivalents of the aforementioned compounds. To detect inhibitory effects on enzymes, we used colorimetric enzyme inhibition assays, and these assays included tyrosinase, α-glucosidase, α-amylase, and cholinesterases. Standard inhibitors (galantamine for cholinesterases, kojic acid for tyrosinase, and acarbose for α-glucosidase and α-amylase) were used as positive controls [49]. The experimental details are given in Supplementary Materials.

### 3.7. Statistical Analysis

A one-way analysis of the variance (ANOVA) was performed to investigate significant differences (*p* < 0.05, Turkey’s post hoc test) for each assay done. Thereafter, the dataset was submitted to *k*-medoids cluster analysis after the distance matrix was visualized for measuring the (dis)similarity between the observations. Euclidean distance measure was used for this purpose. To estimate the optimal number of clusters, the average silhouette method was used. A high average silhouette width indicated a good clustering solution. Finally, the goodness of the *k*-medoids clustering was evaluated by estimating the silhouette coefficient (*S_i_*). A value of *S_i_* close to 1 confirmed a good clustering solution. The statistical analysis was performed by using R 3.5.1 software (R Core Team, Vienna, Austria).

## 4. Conclusions

The present study offers a new perspective in the potential use of *C. hirtus* as a source of bioactive constituents. Fingerprinting showed the different plant constituents and suggested the possibility of extracting significant amounts of different classes of compounds—terpenoids, phytosterols, phenolics—from plant material using methanol as solvent. Notable antioxidant and enzyme inhibitory effects against cholinesterases (AChE and BChE) and tyrosinase were displayed by the extracts. Among the tested solvents, the polar solvents (water and MeOH) were the best solvents for extracting bioactive compounds with antioxidant properties from *C. hirtus,* whilst the MeOH, DCM, and EA were suitable for obtaining biomolecules with enzyme inhibitory potentials, suggesting that multiple constituents in the extracts being soluble in different solvents exerted inhibitory activity on the same enzyme. This suggests a rationale for the use of the overall mixture, which can offer the opportunity to act on the same target compounds with different physicochemical properties. Based on the present findings, *C. hirtus* can be considered as a valuable source of pharmacologically active agents and used for designing phytomedicines. Nonetheless, further investigations are essential to elucidate the mode of action of the antioxidant and the enzyme inhibition activity, toxicity, safety, and bioavailability of the extracts.

## Figures and Tables

**Table 1:** NMR data obtained from 1D and 2D NMR experiments and assignments of constituents identified in *Croton hirtus* extract. COSY, correlation spectroscopy.

Compound No.	Compounds or Class of Compounds Atom Position	δ H	δ c	Correlations in HMBC or COSY
**1**	**Protocatechuic acid** (HMDB0001856)			
	2	7.53	115	167.0 (HMBC); 7.48 (COSY)
	5	6.90	115.4	7.48 (COSY)
	6	7.48	124.7	167.0 (HMBC) 7.53; 6.90 (COSY)
	**Caffeic acid moieties**			
	7	7.59	143.9	167.5; 130.0 (HMBC); 6.45 (COSY)
	8	6.45	115.6	127.4; 7.59 (COSY)
	Aromatic ring protons	7.14–6.80	127.5, 122.3 114.7, 115.6	145.3, 151.0; 127.0, 145; 114 (HMBC)
	**Flavonoids**			
	H 6-8 of glycosidic flavonols	6.10–6.22	98.5–99.7	165–155 99 101 (HMBC)
	Ring B quercetin	7.40–6-23	115.0, 129, 125, 118	150.0, 145.3, 131.0 (HMBC)
	Ring B kaempferol (HMDB0005801)	7.98	130.5	165.0, 130.5 (HMBC); 6.80 (COSY)
		6.80	115.0	115; 7.98 (COSY)
	**Sugar linked to phenolic portions (Flavonol-*O*-glycosides or hydroxycinnamic esters)**			
	Anomeric positions	4.85	109.5	
		4.67	99.8	165.0–160 (HMBC with position 7 of flavonol moieties); 3.30–3.40 (COSY)
		4.43-4.50	101.7	133.8 (HMBC with position 3 of flavonol moieties); 3.18 (COSY)
		4.22	104.7	3.18–3.28 (COSY)
		5.42	103	133.5 (HMBC position 3 flavonol)
	Anomeric positions			
	H-1 (rhamnose)	4.60 brs	99.7	3.18 (COSY)
	H-1 (hexose or pentose)	4.50–4.70 d	100.0–101.1	3.16, 3.23 (COSY)
	H-1 (hexose or pentose)	4.50–4.70	100.0–101.1	3.16, 3.23 (COSY)
	H-6 (hexose) free position	3.30–3.50	60.5	
	H-6 (hexose) glycosidic linked	3.30–3.50	64.5	
	CH bearing ester linkage (from sugar residue)	4.95	73.4	165, 104, 71
	CH bearing ester linkage (from sugar residue)	5.08	71.2	165, 89, 63
	**Megastigmane (aglycone part)**			
	H-2 Icariside B	5.85–5.90	128.7	200, (HMBC)
	CH_2_-6 Icariside B	2.58	54.0	200, 72.0 (HMBC)
	CH_3_ Icariside B	1.02	24.2	77 (HMBC)
	H-7 Corchionoside	5.75	125.7	
	H-8 Corchionoside	5.86	131.9	
	CH3 Corchioside	1.21	19.6	73.0, 125.7 (HMBC)
	**dehydro Ionol derivatives**			
	H3	5.30, 5.2	127.5	141, 59, 36 (HMBC)
	CH_3_ linked to double bond	2.02	20.5	141, 127
	Geminal methyl groups, secondary methyl group of the butanol chain	0.95–1.04	23.9–24.1	36.0 51.0 72.0
	**Fatty acid derivatives**			
	terminal methyl groups	0.93	17.5	40.0, 23.0
	CH2	2.02	19.5	24.2, 30.0
	sp2	5.35–5.40	122.5	

**Table 2:** Identification and quantification of compounds in methanolic extract of *Croton hirtus.* * compared with authentic standard.

Compound No.	Retention Time	[M − H]^−^	Fragments	Formula	Identification and Reference	mg/g
	**Flavonoid glycosides**					
1	16.5	755	593 300(300→271 255 179 151) (271→243 227)	C_33_H_40_O_20_	Quercetin-3-*O*-di(deoxyhexoside)-7-*O*-hexoside	0.61 ± 0.01
2	19.1	755	593 300 (300→271 255 179 151) (271→243 227)	C_36_H_36_O_18_	Quercetin-3-deoxyhexoside-hexoside- deoxyhexoside	0.22 ± 0.01
3	18.9	609	301 300 (301→271 255 179 151) (271→243 227)	C_27_H_30_O_16_	Quercetin-hexoside-deoxyhexoside	1.59 ± 0.05
4	18.5	739	593 577 447 430 285 257	C_33_H_40_O_19_	Kaempferol-7-*O*-hexoside-3-*O*-deoxyhexoside-deoxyhexoside	3.68 ± 0.04
5	19.4	755	609 591 489 300 271 255	C_36_H_36_O_18_	Quercetin-3-deoxyhexoside-hexoside-*p*-coumaroyl	3.64 ± 03.04
6	18.5	595	463 300 271 255 (300→271 255) (271→243 227 215 199)	C_26_H_28_O_16_	Quercetin-3-apiofuranosyl-glucopyranoside	16.13 ± 0.04
7	19.3	609	301 447 285 255	C_27_H_30_O_16_	Rutin*	3.02 ± 0.04
8	20.5	579	447 429 285 (285→255) (255→227 213 211 187)	C_25_H_28_O_15_	Kaempferol-3-*O*-hexosyl pentoside	2.52 ± 0.06
9	19.9	463	301 229 179	C_21_H_20_O_12_	Quercetin-3-*O*-glucoside*	4.21 ± 0.03
10	23.1	623	315 300 299 271 255 243 (315→300 272 255)	C_28_H_30_O_16_	Isorhamnetin-3-*O*-rutinoside*	25.91 ± 0.09
11	21.6	609	315 301 (301→271 255 179 151) (271→243 227)	C_27_H_30_O_16_	Isorhamnetin -3-*O*-hexosyl pentoside	4.93 ± 0.03
12	22.3	447	301 255	C_21_H_20_O_11_	Quercetin-3-*O*-rhamnoside*	1.39 ± 0.04
13	23.5	477	314 285 271	C_21_H_20_O_11_	Isorhamnetin-7-*O*-glucoside*	4.46 ± 0.06
14	25.8	447	314 285 271 (314→300 285 271)	C_21_H_20_O_11_	Isorhamnetin-7-*O*-rhamnoside*	1.97 ± 0.03
15	21.6	593	447 285	C_27_H_30_O_15_	Kaempferol-3-*O*-hexosil-deoxyhexoside	18.00 ± 0.09
16	18.4	329	314 299 271 243 226 199	C_17_H_18_O_7_	Dimethoxy quercetin	4.49 ± 0.02
	**Other phenolics**					
17	2.3	341	179	C_15_H_18_O_9_	Caffeic acid hexoside	4.36 ± 0.04
18	5.8	315	153	C_13_H_16_O_9_	Protocatechuic acid hexoside	5.66 ± 0.03
19	7.8	401	269 161	C_20_H_18_O_9_	Benzyl alcohol hexose pentose	4.04 ± 0.05
20	11.28	487	337 279 261	C_21_H_28_O_13_	Synapoyl pentose-pentose	1.08 ± 0.02
21	17.2	431	261 187 (187→125) (125→97)		Gallic acid benzoic acid derivative	8.84 ± 0.03
22	19.4	769	605 475 315 299	C_35_H_46_O_19_	Leonoside A	10.04 ± 0.02
23	20.4	755	623 593 315 297	C_34_H_44_O_19_	Forsythoside B	6.31 ± 0.05
24	32.6	797	603 474 456 327 167	C_37_H_50_O_19_	Ferruginoside C isomer	7.60 ± 0.05
25	35.5	797	603 474 456 327 167	C_37_H_50_O_19_	Ferruginoside C	14.21 ± 0.08
26	33.9	663	517 485 467	C_31_H_36_O_16_	Feruloyl-coumaroyl saccharose	15.97 ± 0.08
27	36.4	663	517 485 467	C_31_H_36_O_16_	Feruloyl-coumaroyl saccharose	12.21 ± 0.08
28	39.9	663	517 485 467	C_31_H_36_O_16_	Feruloyl-coumaroyl saccharose	11.20 ± 0.06
29	41.5	663	517 485 467	C_31_H_36_O_16_	Feruloyl-coumaroyl saccharose	13.06 ± 0.09
30	44.2	663	517 485 467	C_31_H_36_O_16_	Feruloyl-coumaroyl saccharose	9.06 ± 0.07
	**Hydrophylic Terpenoids (positive electrospray (ESI))**					
31	13.1	433 [M + HCOOH − H]^−^	387.5 223 205 161 153 (153→138–122)	C_19_H_32_O_8_ + CH_2_O_2_	Icariside B5	9.15 ± 0.06
32	13.9	431 [M + HCOOH − H]^−^	385.5 223 205 161 153 (153→138–122)	C_19_H_30_O_8_ + CH_2_O_2_	Corchoionoside C/Roseoside	3.63 ± 0.06
33	22.97	487	355 337 289 279 261 167	C_24_H_40_O_10_	dihydro α ionol-*O*-[arabinosil(1-6) glucoside]	132.72 ± 0.11
34	24.07	487	355 337 289 271	C_24_H_40_O_10_	dihydro β ionol-*O*-[arabinosil(1-6) glucoside]	79.57 ± 0.11
35	53.4	331	295 277 215 185	C_20_H_28_O_4_	Kongensin D	0.72 ± 0.02
	**Phytosterols and terpenoids**		**Positive atmospheric pressure chemical ionization (APCI)**			
		[M − H_2_0 + H]^+^				
36	60.0	397		C_29_H_50_O	β-sitosterol*	48.60 ± 0.14
37	53.8	383		C_28_H_48_O	Campesterol*	3.04 ± 0.08
38	51.3	399		C_29_H_52_O	Stigmastanol*	4.37 ± 0.08

**Table 3:** Extraction yields and total bioactive components in the tested extracts *.

Extracts	Extraction Yields (%)	Total Phenolic Content (mg GAE/g)	Total Flavonoid Content (mg RE/g)
DCM	2.13	24.24 ± 0.90 ^a^	14.37 ± 0.12 ^c^
EA	2.34	22.51 ± 0.52 ^b^	29.28 ± 1.89 ^b^
Infusion	9.95	22.38 ± 0.34 ^b^	12.54 ± 0.32 ^c^
MeOH	13.18	17.96 ± 0.03 ^c^	50.16 ± 2.53 ^a^

* Values expressed are means ± S.D. of three parallel measurements. GAE: Gallic acid equivalent; RE: Rutin equivalent. Different letters indicate significant differences in the extracts (*p* < 0.05).

**Table 4:** Antioxidant properties of the tested extracts *.

Extracts	DPPH (mgTE/g)	ABTS (mgTE/g)	CUPRAC (mgTE/g)	FRAP (mgTE/g)	Phosphomolybdenum (mmol TE/g)	Chelating Ability (mg EDTAE/g)
DCM	22.78 ± 0.52 ^c^	32.32 ± 2.49 ^c^	88.67 ± 1.08 ^a^	26.35 ± 0.09 ^c^	2.70 ± 0.13 ^a^	15.18 ± 1.08 ^b^
EA	23.66 ± 1.14 ^c^	18.59 ± 1.64 ^d^	69.04 ± 0.40 ^c^	24.15 ± 0.21 ^d^	2.33 ± 0.27 ^ab^	18.26 ± 0.22 ^a^
Infusion	41.08 ± 1.65 ^a^	64.84 ± 2.71 ^a^	78.17 ± 0.16 ^b^	45.67 ± 0.86 ^a^	1.46 ± 0.01 ^c^	17.94 ± 0.16 ^a^
MeOH	30.62 ± 0.54 ^b^	42.02 ± 1.11 ^b^	62.50 ± 2.30 ^d^	30.94 ± 0.35 ^b^	1.97± 0.10 ^b^	13.96 ± 0.10 ^b^

* Values expressed are means ± S.D. of three parallel measurements. TE: Trolox equivalent; EDTAE: EDTA equivalent. Different letters indicate significant differences in the extracts (*p* < 0.05).

**Table 5:** Enzyme inhibitory properties of the tested extracts *.

Extracts	AChE Inhibition (mgGALAE/g)	BChE Inhibition (mgGALAE/g)	Tyrosinase Inhibition (mgKAE/g)	Amylase Inhibition (mmolACAE/g)	Glucosidase Inhibition (mmol ACAE/g)
DCM	5.03 ± 0.16 ^a^	16.41 ± 1.68 ^a^	24.39 ± 0.98 ^c^	0.71 ± 0.01 ^ab^	1.68 ± 0.14
EA	4.84 ± 0.19 ^a^	15.86 ± 0.74 ^a^	34.81 ± 2.67 ^b^	0.75 ± 0.03 ^a^	na
Infusion	1.01 ± 0.13 ^c^	14.44 ± 0.34 ^a^	na	0.14 ± 0.01 ^c^	na
MeOH	4.09 ± 0.02 ^b^	16.11 ± 0.25 ^a^	49.83 ± 3.94 ^a^	0.69 ± 0.02 ^b^	na

* Values expressed are means ± S.D. of three parallel measurements. GALAE: Galatamine equivalent; KAE: Kojic acid equivalent; ACAE: Acarbose equivalent. na: not active. Different letters indicate significant differences in the extracts (*p* < 0.05).

## Data Availability

Data is not available from the authors.

## Supplementary Materials

Supplementary materials are available online, S1: Croton methanol extract H-NMR spectrum; Croton methanol extract HSQC-NMR spectrum enlargement of aromatic portion; Croton methanol extract HMBC spectrum; Croton methanol extract DOSY spectrum; S2: LC-MS in negative ion mode showing the two peaks and relative mass spectra ascribable to Icariside B5 ([M + HCOOH – H]- ion at *m*/*z* 433) and Corchionoside([M + HCOOH – H]- ion at *m*/*z* 431); S3: LC-MS in negative ion mode showing the two peaks and relative mass spectra ascribable to dihydro α ionol-*O*-[arabinosil(1-6) glucoside] and the dihydro β ionol-*O*-[arabinosil(1-6) glucoside]. Ions are detected as [M + HCOOH – H]-and also as [M – H]- as visible in figure.

## Sample Availability

Samples of the compounds are not available from the authors.
