# Supplementary material for: Evaluation of Antioxidant and Enzyme Inhibition Properties of Croton hirtus L’Hér. Extracts Obtained with Different Solvents
Source: Molecules. 2021 Mar 28;26(7):1902. doi: 10.3390/molecules26071902 (PMC8038089; doi:10.3390/molecules26071902)
Supplement: Supplementary file 1 [file molecules-26-01902-s001.pdf]

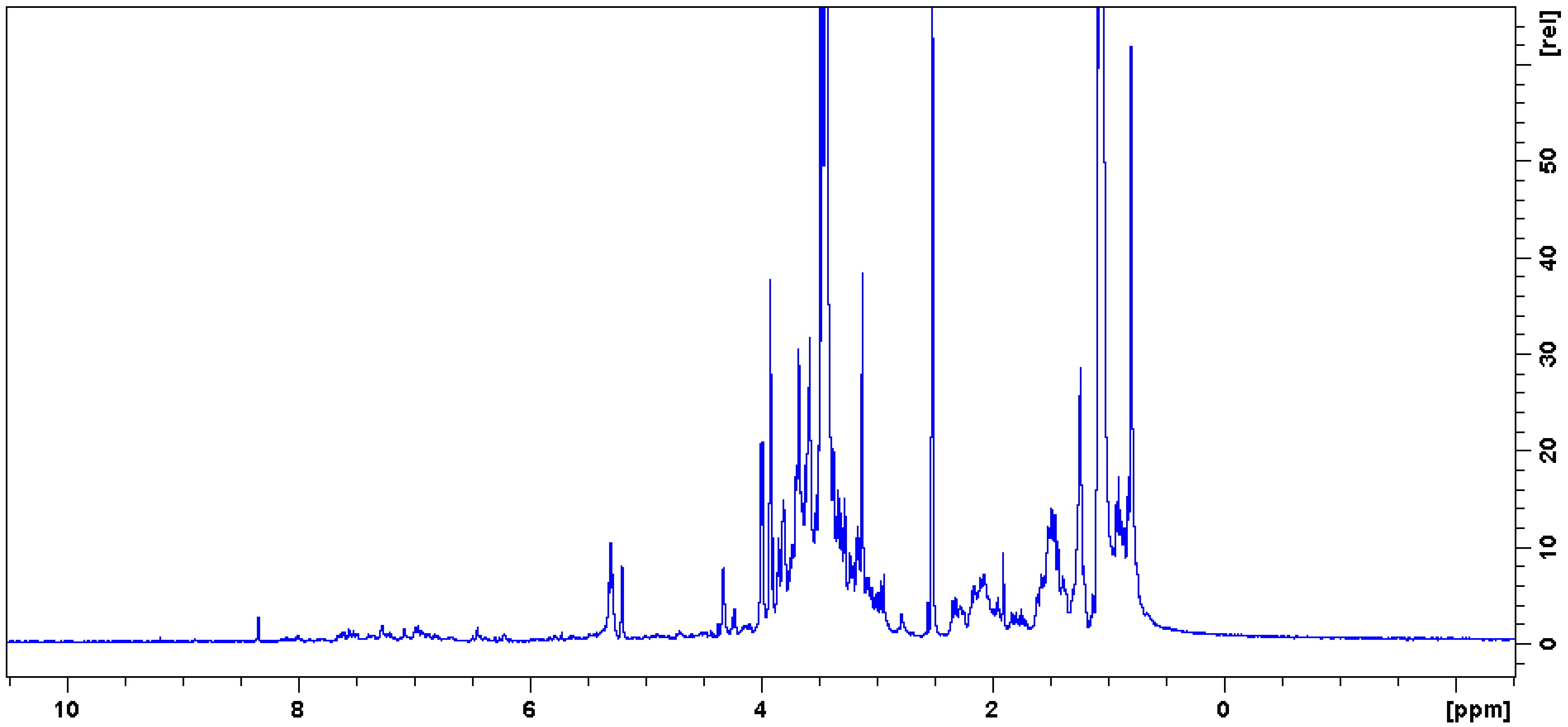

S1: Croton methanol extract H-NMR spectrum

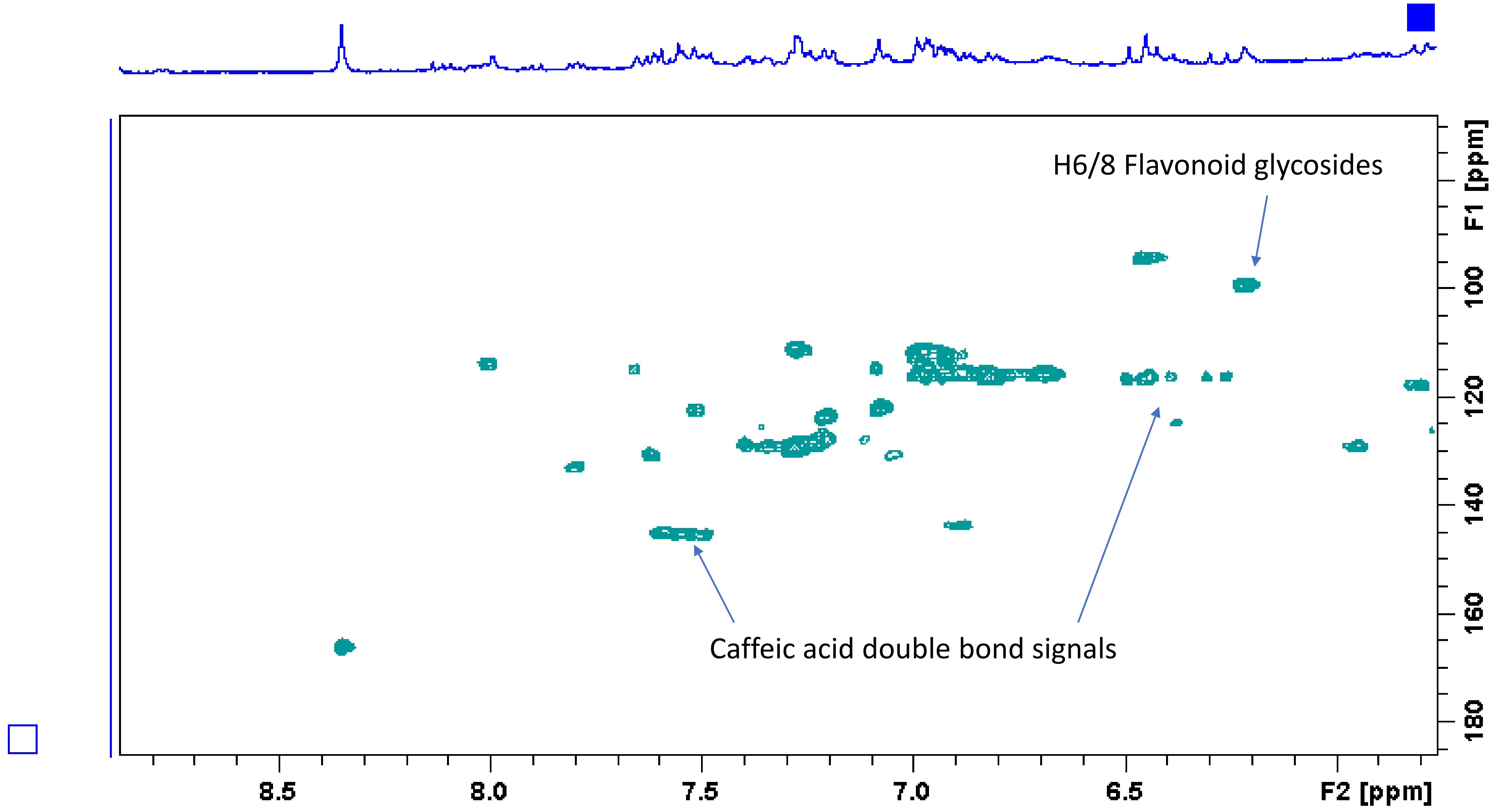

S1: Croton methanol extract HSQC-NMR spectrum enlargment of aromatic portion

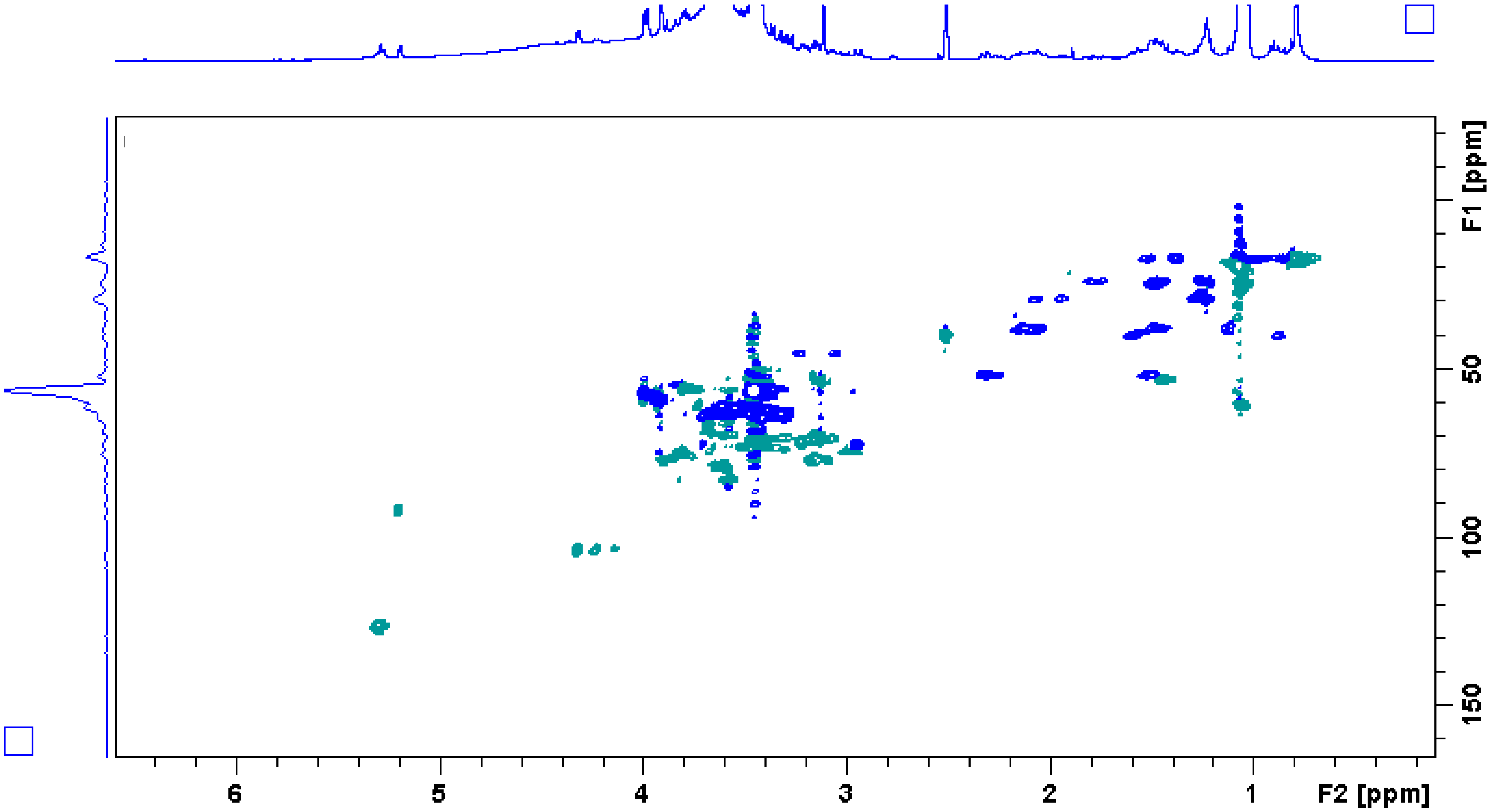

S1: Croton methanol extract HSQC-NMR spectrum ppm range 0-6.5

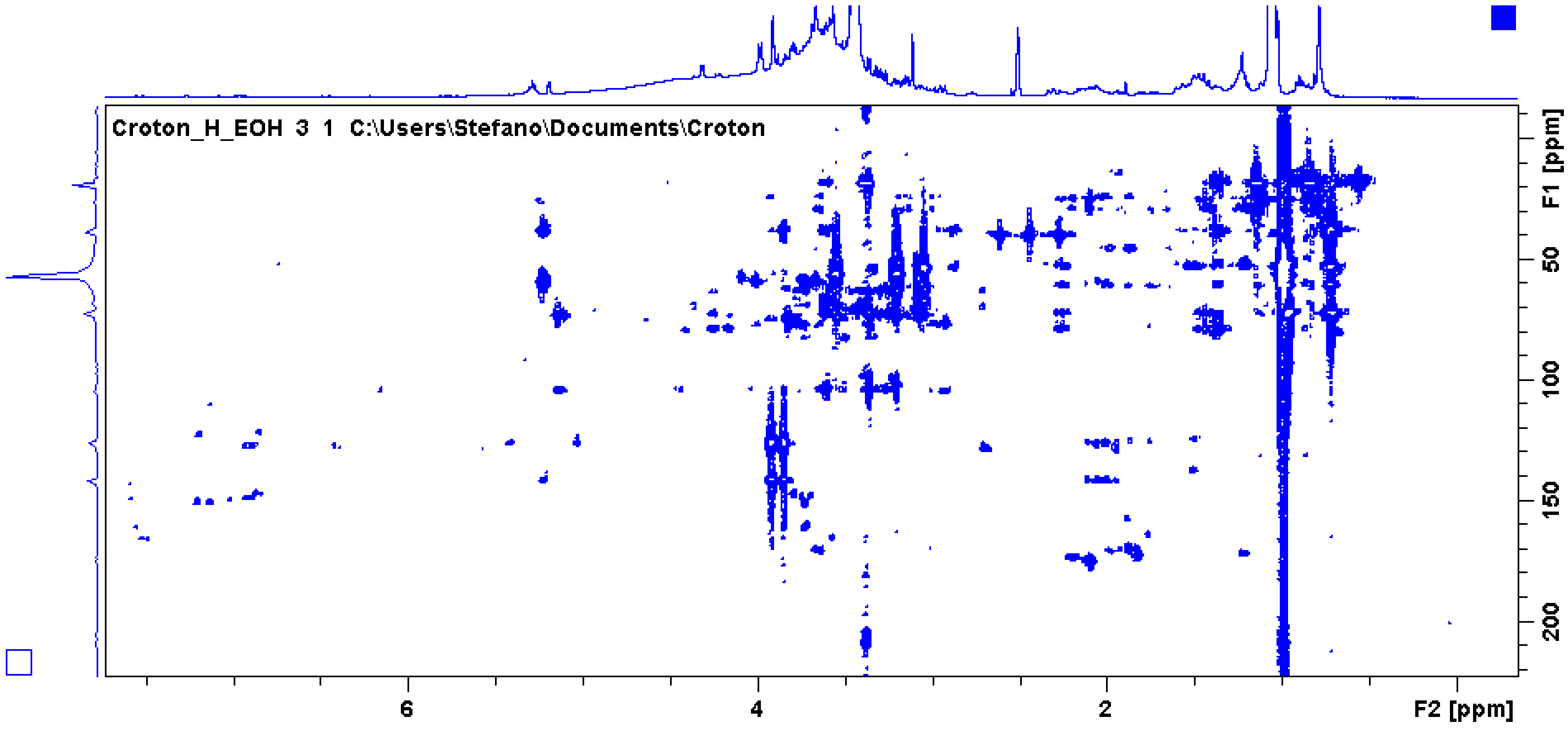

S1: Croton methanol extract HMBC spectrum

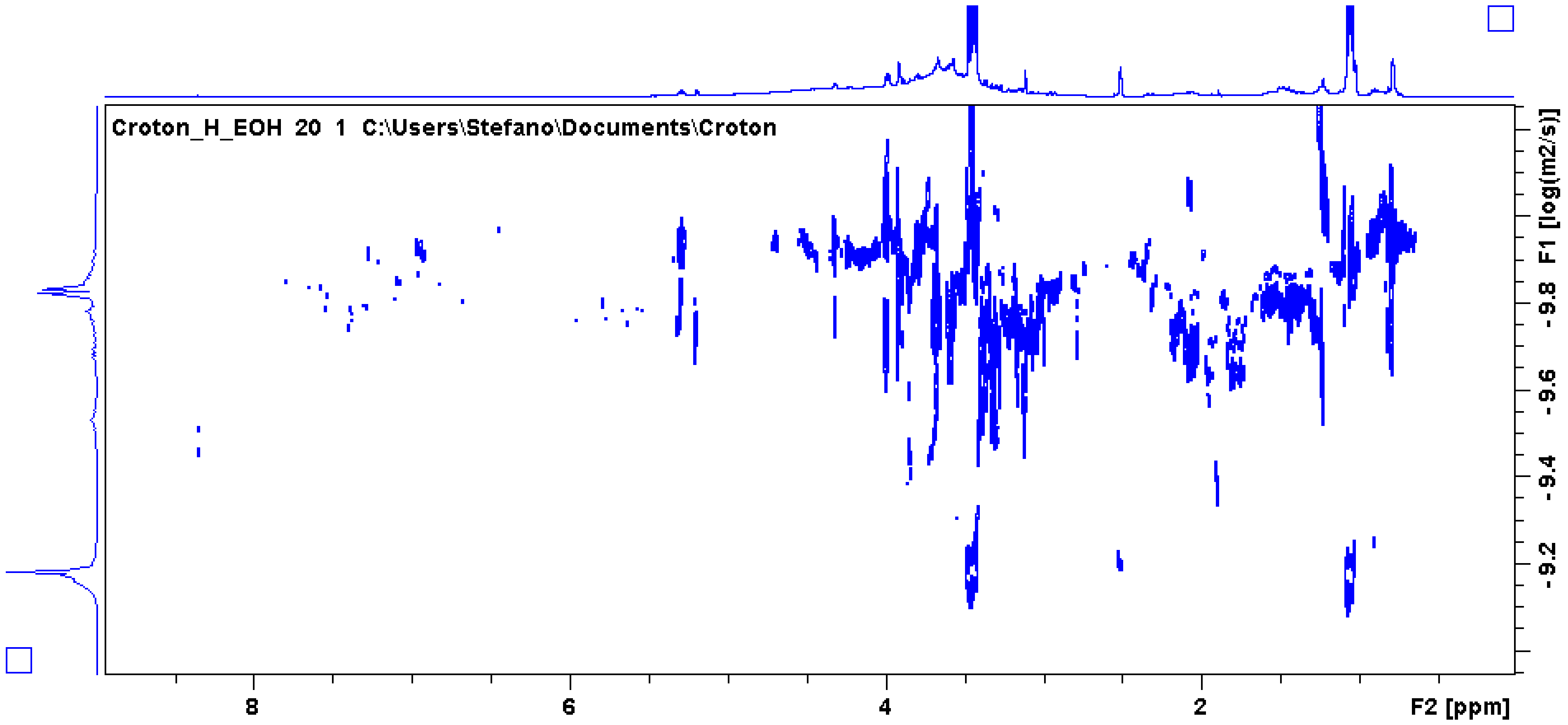

S1: Croton methanol extract DOSY spectrum

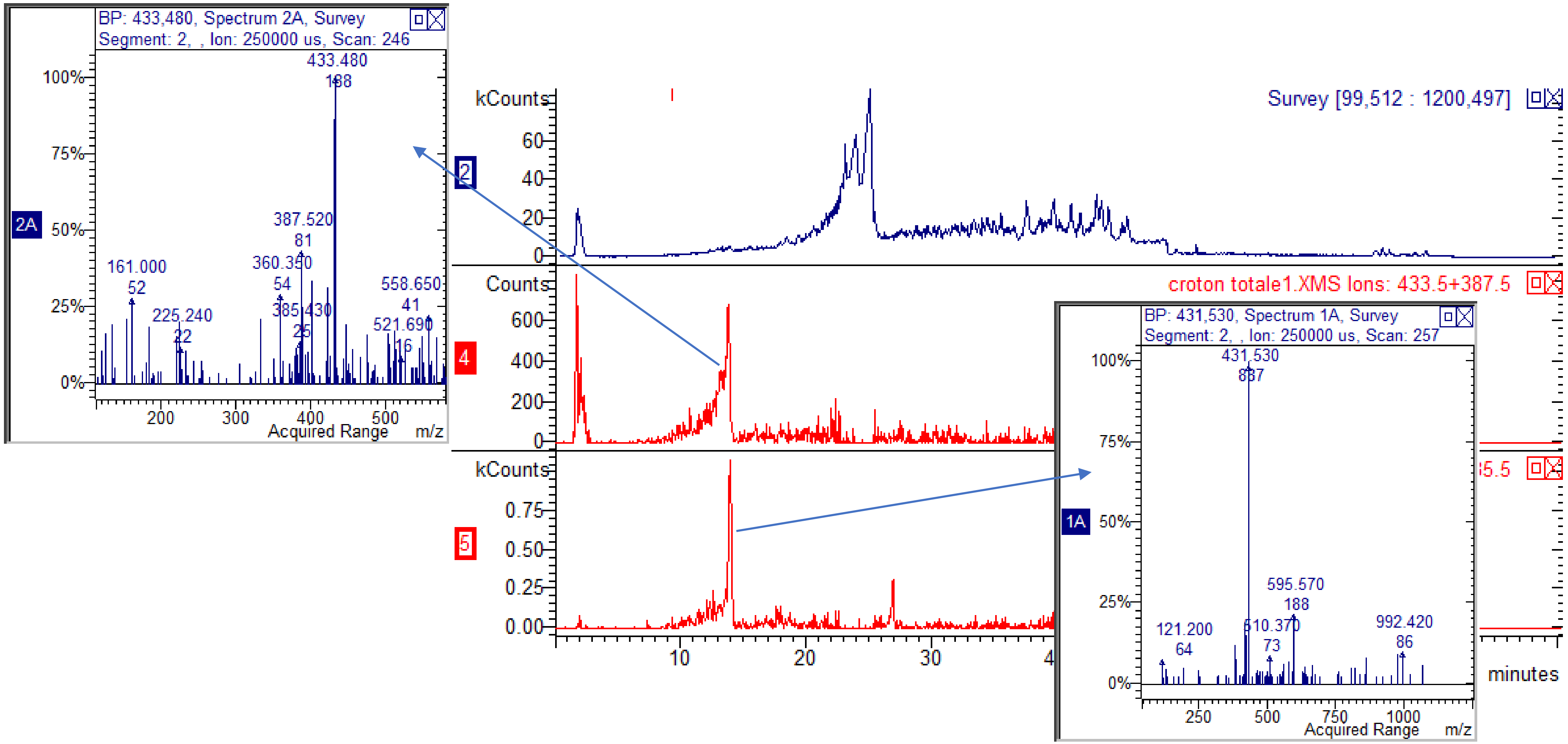

S2: LC-MS in negative ion mode showing the two peaks and relative mass spectra ascribable to Icariside B5 ([M+HCOOH-H]- ion at m/z 433) and Corchionoside ([M+HCOOH-H]- ion at m/z 431)

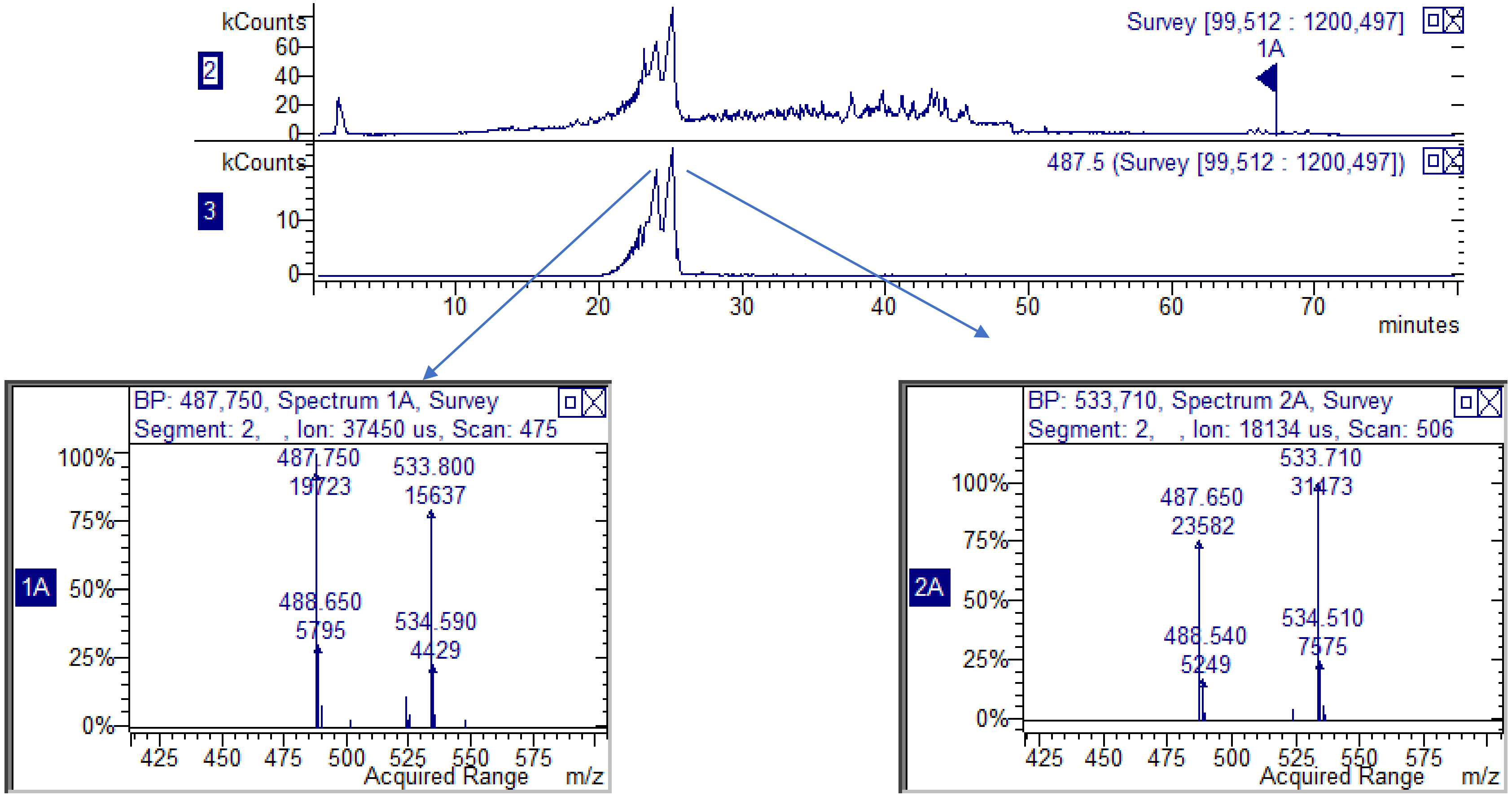

S3: LC-MS in negative ion mode showing the two peaks and relative mass spectra ascribable to dihydro α ionol-O-[arabinosil(1-6) glucoside] and the dihydro β ionol-O-[arabinosil(1-6) glucoside]. Ions are detected as [M+HCOOH-H]- and also as [M-H]- as visible in figure
